# Supplementary material for: Impact of Anti-IL5 Therapies on Patients with Severe Uncontrolled Asthma and Possible Predictive Biomarkers of Response: A Real-Life Study
Source: Int J Mol Sci. 2023 Jan 19;24(3):2011. doi: 10.3390/ijms24032011 (PMC9917054; doi:10.3390/ijms24032011)
Supplement: Supplementary file 1 [file ijms-24-02011-s001.zip › Table S1 .pdf]

Table S1: Predictors of oral corticosteroid bursts reduction at 12 months of mepolizumab treatment in patients with severe uncontrolled asthma (bivariate analysis).

	**Response to oral corticosteroid reduction**						
Independent variable	N	Unsatisfactory	Satisfactory	p-value	Reference category	OR	$CI_{95\%}$
Age	89	53.69 ± 13.74	56.88 ± 12.83	0.284	-	-	-
Sex							
Female	58	18 (31)	40 (69)	0.670	-	-	-
Male	31	11 (35.5)	20 (64.5)				
BMI							
Underweight	4	1 (25)	3 (75)	0.171	-	-	-
Normal weight	19	3 (15.8)	16 (84.2)				
Overweight	38	12 (31.6)	26 (68.4)				
Obesity	28	13 (46.4)	15 (53.6)				
Tobacco consumption							
Non smoker	74	26 (35.1)	48 (64.9)	0.254	-	-	-
Former smoker	15	3 (20)	12 (80)				
Current smoker	0	0	0				
Previous respiratory disease							
Yes	38	13 (34.2)	25 (65.8)	0.778	-	-	-
No	51	16 (31.4)	35 (686)				
Polyps							
Yes	38	13 (34.2)	25 (65.8)	0.778	-	-	-
No	51	16 (31.4)	35 (686)				
Allergies							
Yes	44	12 (27.3)	32 (72.7)	0.290	-	-	-
No	45	17 (37.8)	28 (62.2)				
GERD							
Yes	35	14 (40)	21 (60)	0.230	-	-	-
No	54	15 (27.8)	39 (72.2)				
SAHS							

Yes	16	5 (31.2)	11 (68.8)	0.900	-	-	-
No	73	24 (32.9)	49 (67.1)				
COPD							
Yes	14	2 (14.3)	12 (85.7)	0.112	-	-	-
No	75	27 (36)	48 (64)				
Years with AE	89	4 [3-7]	7.5 [3.8-11]	0.087		-	-
ICS (mg/day)	89	500 [184-800]	200 [184-640]	0.052	-	0.99	[0.99-1]
Bursts of OCS per year	89	2 [1-4]	2 [0-4.5]	0.616		-	-
Yes	65	25 (38.5)	40 (61.5)	0.065	Si	2.97	[0.98-11.14]
No	23	4 (17.4)	19 (82.61)				
Maintenance OCS	89	0 [0-0]	0 [0-0]	0.58			
Yes	6	3 (50)	3 (50)	0.346*	-	-	-
No	83	26 (31.3)	57 (68.7)				
Baseline FEV1 (%)	85	76.5 ± 24.5	68.47 ± 22.98	0.145	-	-	-
<80	60	17 (28.3)	43 (71.7)	0.161	-	-	-
>80	25	11 (44)	14 (56)				
Baseline ACT	28	12.5 [11-13]	12.5 [9-19.3]	0.310		-	-
Exacerbation in previous year	89	2 [1-3]	0 [0-1.5]	<0.001	-	0.51	[0.35-0.71]
Yes	53	24 (45.3)	29 (54.7)	0.003	Si	4.97	[1.78-16.34]
No	35	5 (14.3)	30 (85.7)				
Basal blood eosinophils (cell/mcl)	88	680 [290-1000]	610 [335-880]	0.281	-	-	-
Baseline IgE (IU/Ml)	54	111 [55.5-509.2]	114.1 [25.8-267]	0.809		-	-
Years with mepolizumab	89	3 [1-4]	2 [1-4]	0.813		-	-
Previous BT							
COPD	23	9 (39.1)	14 (60.9)	0.437	-	-	-
Yes	66	20 (33.3)	46 (69.7)				

BMI, body mass index; GERD, gastro-oesophageal reflux disease; SAHS, sleep apnoea-hypopnoea syndrome; COPD, chronic obstructive pulmonary disease; EC, eosinophilic asthma; ICS, inhaled corticosteroids; OCS, oral corticosteroids; FEV1, peak expiratory volume in the first second of forced expiration; ACT, Asthma Control Test; IgE, immunoglobulin E; BT, biological therapy. OR, Odds ratio; CI95%, 95% confidence interval.

Unsatisfactory: There is no greater than 50% reduction in OCS bursts, nor absence of OCS bursts.; Satisfactory: reduction of at least 50% or absence of OCS bursts.

*Fisher's exact test
