# Supplementary material for: Impact of Anti-IL5 Therapies on Patients with Severe Uncontrolled Asthma and Possible Predictive Biomarkers of Response: A Real-Life Study
Source: Int J Mol Sci. 2023 Jan 19;24(3):2011. doi: 10.3390/ijms24032011 (PMC9917054; doi:10.3390/ijms24032011)
Supplement: Supplementary file 1 [file ijms-24-02011-s001.zip › Table S2.pdf]

Table S2: Predictors of reduction in maintenance oral corticosteroids at 12 months of mepolizumab treatment in patients with severe uncontrolled asthma (bivariate analysis).

	**Response to oral corticosteroid reduction**						
Independent variable	N	Unsatisfactory	Satisfactory	p-value	Reference category	OR	$CI_{95\%}$
Age	89	63 ± 2.83	55.68 ± 13.25	0.434	-	-	-
Sex							
Female	58	1 (1.7)	57 (98.3)	1*	-	-	-
Male	31	1 (3.2)	30 (96.8)				
BMI							
Underweight	4	0 (0)	4 (100)	1*	-	-	-
Normal weight	19	0 (0)	19 (100)				
Overweight	39	1 (2.6)	38 (97.4)				
Obesity	27	1 (3.7)	26 (96.3)				
Tobacco consumption							
Non smoker	74	1 (1.4)	73 (98.6)	0.31*	-	-	-
Former smoker	15	1 (6.7)	14 (93.3)				
Current smoker	0	0 (0)	0 (0)				
Previous respiratory disease							
Yes	38	1 (2.6)	37 (9.4)	1*	-	-	-
No	51	1 (2)	50 (98)				
Polyps							
Yes	38	2 (5.3)	36 (94.7)	0.18*	-	-	-
No	51	0 (0)	51 (100)				
Allergies							
Yes	44	1 (2.3)	43 (97.7)	1*	-	-	-
No	45	1 (2.2)	44 (97.8)				
GERD							
Yes	35	0 (0)	35 (100)	0.517*	-	-	-
No	54	2 (3.7)	52 (96.3)				
SAHS							

Yes	16	1 (6.2)	15 (93.8)	0.329*	-	-	-
No	73	1 (1.4)	72 (98.6)				
COPD							
Yes	14	0 (0)	14 (100)	1*	-	-	-
No	75	2 (2.7)	73 (97.3)				
Years with AE	89	5 [4-6]	6 [3-10.5]	0.555	-	-	-
ICS (mg/day)	89	550 [525-575]	200 [184-640]	0.614	-		
Bursts of OCS per year	89	12 [12-12]	2 [0-4]	0.997	-	-	-
Yes	65	2 (3.1)	63 (96.9)	1*	-	-	-
No	23	0 (0)	23 (100)				
Maintenance OCS	89	10 [7.5-12.5]	0 [0-0]	0.008	-	0.73	0.55-0.91
Yes	6	4 (66.7)	2 (33.3)	0.004*	Si	$3.16e^{9}$	0-NA
No	83	0 (0)	83 (100)				
Baseline FEV1 (%)	85	91.5 ± 0.71	70.63 ± 23.72	0.241	-	-	-
<80	60	0 (0)	60 (100)	0.084*	>80	$2.02e^{8}$	4.05e-^{262}-NA
>80	25	2 (8)	23 (92)				
Baseline ACT	28	-	12.5 [9-15.6]	1	-	-	-
Exacerbation in previous year	89	3.5 [2.8-4.3]	1 [0-2]	0.086	-	0.5	0.19-1.09
Yes	53	2 (3.8)	51 (96.2)	0.515*	-	-	-
No	35	0 (0)	35 (100)				
Basal blood eosinophils (cell/mcl)	89	680 [683-688]	635 [330-915]	0.930	-	-	-
Baseline IgE (IU/Ml)	54	95 [95-95]	114.1 [29-283]	0.695	-	-	-
Years with mepolizumab	89	4 [3.5-4.5]	2 [1-4]	0.194	-	-	-
Previous BT							
Yes	23	0 (0)	23 (100)	1*	-	-	-
No	66	2 (3)	64 (97)				

BMI, body mass index; GERD, gastro-oesophageal reflux disease; SAHS, sleep apnoea-hypopnoea syndrome; COPD, chronic obstructive pulmonary disease; EC, eosinophilic asthma; ICS, inhaled corticosteroids; OCS, oral corticosteroids; FEV1, peak expiratory volume in the first second of forced expiration; ACT, Asthma Control Test; IgE, immunoglobulin E; BT, biological therapy. OR, Odds ratio; CI95%, 95% confidence interval.

Unsatisfactory: no more than 50% reduction in maintenance OCS dose, or complete elimination of maintenance OCS; Satisfactory: at least 50% reduction in maintenance OCS dose or elimination of maintenance OCS dose.

*Fisher's exact test
