# Supplementary material for: Impact of Anti-IL5 Therapies on Patients with Severe Uncontrolled Asthma and Possible Predictive Biomarkers of Response: A Real-Life Study
Source: Int J Mol Sci. 2023 Jan 19;24(3):2011. doi: 10.3390/ijms24032011 (PMC9917054; doi:10.3390/ijms24032011)
Supplement: Supplementary file 1 [file ijms-24-02011-s001.zip › Table S3.pdf]

Table S3: Predictors of lung function improvement at 12 months of mepolizumab treatment in patients with severe uncontrolled asthma (bivariate analysis).

	**Response to improved lung function**						
Independent variable	N	Unsatisfactory	Satisfactory	p-value	Reference category	OR	$CI_{95\%}$
Age	79	51.54 ± 13.15	59.05 ± 11.34	0.013	-	1.05	[1.01-1.1]
Sex							
Female	53	27 (50.9)	26 (49.1)	0.808	-	-	-
Male	26	14 (53.8)	12 (46.2)				
BMI							
Underweight	3	1 (33.3)	2 (66.7)	0.305*	-	-	-
Normal weight	16	7 (43.8)	9 (56.2)				
Overweight	33	15 (45.5)	18 (54.5)				
Obesity	27	18 (66.7)	9 (33.3)				
Tobacco consumption							
Non smoker	-	-	-	0.652	-	-	-
Former smoker	15	7 (46.7)	8 (53.3)				
Current smoker	64	34 (53.1)	30 (46.9)				
Previous respiratory disease							
Yes	34	14 (41.2)	20 (58.8)	0.097	-	-	-
No	45	27 (60)	18 (40)				
Polyps							
Yes	35	16 (45.7)	19 (54.3)	0.327	-	-	-
No	44	25 (56.8)	19 (43.2)				
Allergies							
Yes	41	20 (48.8)	21 (51.2)	0.565	-	-	-
No	38	21 (55.3)	17 (44.7)				
GERD							
Yes	32	19 (594)	13 (40.6)	0.273	-	-	-
No	47	22 (46.8)	25 (53.2)				

SAHS							
Yes	14	11 (78.6)	3 (21.4)	0.028	Yes	4.28	[1.20-20.23]
No	65	30 (46.2)	35 (53.8)				
COPD							
Yes	11	4 (36.4)	7 (63.6)	0.266	-	-	-
No	68	37 (54.4)	31 (45.6)				
Years with AE	79	6 [3-11]	7 [2.3-10]	0.641	-	-	-
ICS (mg/day)	79	320 [184-640]	184 [184-400]	0.259	-	-	-
Bursts of OCS per year	79	2 [0-4]	3 [1-5]	0.416	-	-	-
Yes	57	27 (47.4)	30 (52.6)	0.195	-	-	-
No	22	14 (63.6)	8 (36.4)				
Maintenance OCS	79	0 [0-0]	0 [0-0]	0.789	-	-	-
Yes	5	3 (60)	2 (40)	1*	-	-	-
No	74	38 (51.4)	36 (48.6)				
Baseline FEV1 (%)	79	85.53 ± 19.55	55.92 ± 14.77	<0.001	-	0.91	[0.86-0.94]
<80	54	19 (35.2)	35 (64.8)	<0.001	>80	20.26	[5.21-135.30]
>80	24	22 (91.7)	2 (8.3)				
Baseline ACT	25	12 [7.5-15]	12 [9-19]	0.656	-	-	-
Exacerbation in previous year	79	1 [0-2]	1 [0-2]	0.526	-	-	-
Yes	49	25 (51)	24 (49)	0.842	-	-	-
No	30	16 (53.3)	14 (46.7)				
Basal blood eosinophils (cell/mcl)	79	490 [260-800]	745 [540-990]	0.828	-	-	-
Baseline IgE (IU/Ml)	50	82.5 [26.6-269.3]	120.7 [40.5-279.5]	0.713	-	-	-
Years with mepolizumab	79	2 [1-4]	2 [1-4]	0.241	-	-	-
Previous BT							
Yes	20	11 (55)	9 (45)	0.748	-	-	-
No	59	30 (50.8)	29 (49.2)				

BMI, body mass index; GERD, gastro-oesophageal reflux disease; SAHS, sleep apnoea-hypopnoea syndrome; COPD, chronic obstructive pulmonary disease; EC, eosinophilic asthma; ICS, inhaled corticosteroids; OCS, oral corticosteroids; FEV1, peak expiratory volume in the first second of forced expiration; ACT, Asthma Control Test; IgE, immunoglobulin E; BT, biological therapy. OR, Odds ratio; CI95%, 95% confidence interval.

Unsatisfactory: does not increase FEV1 in 10%; Satisfactory: FEV1 increase of at least 10%.

*Fisher's exact test
