# Supplementary material for: Impact of Anti-IL5 Therapies on Patients with Severe Uncontrolled Asthma and Possible Predictive Biomarkers of Response: A Real-Life Study
Source: Int J Mol Sci. 2023 Jan 19;24(3):2011. doi: 10.3390/ijms24032011 (PMC9917054; doi:10.3390/ijms24032011)
Supplement: Supplementary file 1 [file ijms-24-02011-s001.zip › Table S4.pdf]

Table S4: Predictors of exacerbation reduction at 12 months of mepolizumab treatment in patients with severe uncontrolled asthma (bivariate analysis).

	**Response to exacerbation reduction**						
Independent variable	N	Unsatisfactory	Satisfactory	p-value	Reference category	OR	$CI_{95\%}$
Age	89	54.8 ± 13.35	55.97 ± 13.2	0.789	-	-	-
Sex							
Female	58	9 (15.5)	49 (84.5)	0.080	-	-	-
Male	31	1 (3.2)	30 (96.8)				
BMI							
Underweight	4	1 (25)	3 (75)	0.342*	-	-	-
Normal weight	19	1 (5.3)	18 (94.7)				
Overweight	38	3 (7.9)	35 (92.1)				
Obesity	28	5 (17.9)	23 (82.1)				
Tobacco consumption							
Non smoker	74	9 (12.2)	65 (87.8)	0.539	-	-	-
Former smoker	15	1 (6.7)	14 (93.3)				
Current smoker	-	-	-				
Previous respiratory disease							
Yes	38	6 (15.8)	32 (84.2)	0.240	-	-	-
No	51	4 (7.8)	47 (92.2)				
Polyps							
Yes	38	4 (10.5)	34 (89.5)	0.855	-	-	-
No	51	6 (11.8)	45 (88.2)				
Allergies							
Yes	44	7 (15.9)	37 (84.1)	0.168	-	-	-
No	45	3 (6.7)	42 (93.3)				
GERD							
Yes	35	3 (8.6)	32 (91.4)	0.522	-	-	-
No	54	7 (13)	47 (87)				
SAHS							

Yes	16	2 (12.5)	14 (87.5)	0.8597	-	-	-
No	73	8 (11)	65 (89)				
COPD							
Yes	14	2 (14.3)	12 (85.7)	0.6939	-	-	-
No	75	8 (10.7)	67 (89.3)				
Years with AE	89	8 [3.3-10]	6 [3-10.5]	0.9294	-	-	-
ICS (mg/day)	89	184 [184-5550]	285 [184-640]	0.546	-	-	-
Bursts of OCS per year	89	1 [1-3.8]	2 [0-4]	0.411	-	-	-
Yes	65	8 (12.3)	57 (87.7)	0.639	-	-	-
No	23	2 (8.7)	21 (91.3)				
Maintenance OCS	89	0 [0-0]	0 [0-0]	0.992	-	-	-
Yes	6	0 (0)	6 (100)	0.367	-	-	-
No	83	10 (12)	73 (88)				
Baseline FEV1 (%)	85	59.9 ± 14.84	72.75 ± 24.2	0.0887	-	-	-
<80	60	9 (15)	51 (85)	0.1515	-	-	-
>80	25	1 (4)	24 (96)				
Baseline ACT	28	13 [12.8-13]	12 [8.8-18.3]	0.874	-	-	-
Exacerbation in previous year	89	2 [1-3]	1 [0-2]	0.0828	-	-	-
Yes	35	9 (17)	44 (83)	0.0410	Yes	6.95	[1.22-131.40]
No	35	1 (2.9)	34 (97.1)				
Basal blood eosinophils (cell/mcl)	89	420 [292.5-922.5]	660 [342.5-892.5]	0.6339	-	-	-
Baseline IgE (IU/Ml)	55	354.4 [56.1-647]	111 [29-267]	0.689	-	-	-
Years with mepolizumab	89	2.5 [1.1-3]	2 [1-4]	0.9534	-	-	-
Previous BT							
Yes	23	5 (21.7)	18 (78.3)	0.064	Yes	3.38	[0.86-13.50]
No	66	5 (7.6)	61 (92.4)				

BMI, body mass index; GERD, gastro-oesophageal reflux disease; SAHS, sleep apnoea-hypopnoea syndrome; COPD, chronic obstructive pulmonary disease; EC, eosinophilic asthma; ICS, inhaled corticosteroids; OCS, oral corticosteroids; FEV1, peak expiratory volume in the first second of forced expiration; ACT, Asthma Control Test; IgE, immunoglobulin E; BT, biological therapy. OR, Odds ratio; CI95%, 95% confidence interval.

Unsatisfactory: no reduction of 50% of exacerbations, or absence; Satisfactory: reduction of at least 50% of exacerbations or absence of exacerbations.

*Fisher's exact test
