# Supplementary material for: Impact of Anti-IL5 Therapies on Patients with Severe Uncontrolled Asthma and Possible Predictive Biomarkers of Response: A Real-Life Study
Source: Int J Mol Sci. 2023 Jan 19;24(3):2011. doi: 10.3390/ijms24032011 (PMC9917054; doi:10.3390/ijms24032011)
Supplement: Supplementary file 1 [file ijms-24-02011-s001.zip › Table S5.pdf]

Table S5: Predictors of oral corticosteroid bursts reduction at 12 months of benralizumab treatment in patients with severe uncontrolled asthma (bivariate analysis).

	**Response to oral corticosteroid reduction**						
Independent variable	N	Unsatisfactory	Satisfactory	p-value	Reference category	OR	$CI_{95\%}$
Age	57	59.86 ± 11.48	57.51 ± 15.9	0.543	-	-	-
Sex							
Female	37	11 (29.7)	26 (70.3)	0.061	Male	2.88	[0.94-9.2]
Male	20	11 (55)	9 (45)				
BMI							
Underweight	0	0	0	0.852	-	-	-
Normal weight	12	5 (41.7)	7 (58.3)				
Overweight	19	8 (42.1)	11 (57.9)				
Obesity	26	9 (34.6)	17 (65.4)				
Tobacco consumption							
Non smoker	1	0 (0)	1 (100)	0.691*	-	-	-
Former smoker	14	5 (35.7)	9 (64.3)				
Current smoker	42	17 (40.5)	25 (59.5)				
Previous respiratory disease							
Yes	26	11 (42.3)	15 (57.7)	0.598	-	-	-
No	31	11 (35.5)	20 (64.5				
Polyps							
Yes	22	10 (45.5)	12 (54.5)	0.399	-	-	-
No	35	12 (34.3)	23 (65.7)				
Allergies							
Yes	35	10 (28.6)	25 (71.4)	0.050	No	3.0	[1-9.43]
No	22	12 (54.5)	10 (45.5)				
GERD							
Yes	23	10 (43.5)	13 (56.5)	0.534	-	-	-
No	34	12 (35.3)	22 (64.7)				
SAHS							

Yes	10	4 (40)	6 (60)	0.92	-	-	-
No	47	18 (38.3)	29 (61.7)				
COPD							
Yes	11	7 (63.6)	4 (36.4)	0.058	Yes	3.62	[0.94-15.68]
No	46	15 (32.6)	31 (67.4)				
Years with AE	57	7.5 [5-10]	6 [3.5-10]	0.672	-	-	-
ICS (mg/day)	57	184 [184-800]	184 [184-550]	0.457	-	-	-
Bursts of OCS per year	57	3 [1.3-5.8]	2 [1-3.5]	0.015	-	0.75	[0.57-0.92]
Yes	50	22 (44)	28 (56)	0.036*	Yes	$3.34e^{7}$	[$2.91e^{-51}$-NA]
No	7	0 (0)	7 (100)				
Maintenance OCS	57	0 [0-0]	0 [0-0]	0.993	-	-	-
Yes	5	5 (100)	0 (0)	0.006*	-	-	-
No	52	17 (32.7)	35 (67.3)				
Baseline FEV1 (%)	57	69.55 ± 26.63	72.97 ± 19.81	0.574	-	-	-
<80	38	16 (42.1)	22 (57.9)	0.442	-	-	-
>80	19	6 (31.6)	13 (68.4)				
Baseline ACT	27	12 [10-18]	13 [9.8-14.8]	0.618	-	-	-
Exacerbation in previous year	57	0 [0-1]	0 [0-1]	0.889	-	-	-
Yes	26	9 (34.6)	17 (65.4)	0.572	-	-	-
No	31	13 (41.9)	18 (58.1)				
Basal blood eosinophils (cell/mcl)	57	240 [92.5-425]	490 [360-600]	0.007	-	1.01	[1.00-1.01]
Baseline IgE (IU/Ml)	45	187.9 [60.7-710-2]	114 [50-790]	0.708	-	-	-
Years with benralizumab	57	1.5 [1-2.8]	2 [1-3]	0.297	-	-	-
Previous BT							
Yes	19	9 (47.4)	10 (52.6)	0.336	-	-	-
No	38	13 (34.2)	25 (65.8)				

BMI, body mass index; GERD, gastro-oesophageal reflux disease; SAHS, sleep apnoea-hypopnoea syndrome; COPD, chronic obstructive pulmonary disease; EC, eosinophilic asthma; ICS, inhaled corticosteroids; OCS, oral corticosteroids; FEV1, peak expiratory volume in the first second of forced expiration; ACT, Asthma Control Test; IgE, immunoglobulin E; BT, biological therapy. OR, Odds ratio; CI95%, 95% confidence interval.

Unsatisfactory: There is no greater than 50% reduction in OCS bursts, nor absence of OCS bursts.; Satisfactory: reduction of at least 50% or absence of OCS bursts.

*Fisher's exact test
