# Supplementary material for: Impact of Anti-IL5 Therapies on Patients with Severe Uncontrolled Asthma and Possible Predictive Biomarkers of Response: A Real-Life Study
Source: Int J Mol Sci. 2023 Jan 19;24(3):2011. doi: 10.3390/ijms24032011 (PMC9917054; doi:10.3390/ijms24032011)
Supplement: Supplementary file 1 [file ijms-24-02011-s001.zip › Table S6.pdf]

Table S6: Predictors of reduction in maintenance oral corticosteroids at 12 months of benralizumab treatment in patients with severe uncontrolled asthma (bivariate analysis).

	**Response to oral corticosteroid reduction**						
Independent variable	N	Unsatisfactory	Satisfactory	p-value	Reference category	OR	$CI_{95\%}$
Age	57	56 ± 15.4	58.7 ± 14.3	0.69	-	-	-
Sex							
Female	37	2 (5.4)	35 (94.6)	0.332*	-	-	-
Male	20	3 (15)	17 (85)				
BMI							
Underweight	0	0 (0)	0 (0)	0.705*	-	-	-
Normal weight	12	0 (0)	12 (100)				
Overweight	25	3 (12)	22 (88)				
Obesity	20	2 (10)	18 (90)				
Tobacco consumption							
Non smoker	42	4 (9.5)	38 (90.5)	1*	-	-	-
Former smoker	14	1 (7.1)	13 (92.9)				
Current smoker	1	0 (0)	1 (100)				
Previous respiratory disease							
Yes	26	3 (11.5)	23 (88.5)	0.651*	-	-	-
No	31	2 (6.5)	29 (93.5)				
Polyps							
Yes	22	3 (13.6)	19 (86.4)	0.364*	-	-	-
No	35	2 (5.7)	33 (94.3)				
Allergies							
Yes	35	1 (2.9)	34 (97.1)	0.067*	-	-	-
No	22	4 (18.2)	18 (81.8)				
GERD							
Yes	23	3 (13)	20 (87)	0.384*	-	-	-
No	34	2 (5.9)	32 (94.1)				
SAHS							

Yes	10	1 (10)	9 (90)	1*	-	-	-
No	47	4 (8.5)	43 (91.5)				
COPD							
Yes	11	0 (0)	11 (100)	0.571*	-	-	-
No	46	41 (89.1)	5 (10.9)				
Years with AE	57	7 [5-9]	7 [4-10]	0.929	-	-	-
ICS (mg/day)	57	184 [184-1000]	184 [184-610]	0.499	-		
Bursts of OCS per year	57	6 [3-12]	2 [1-4]	0.014	-	0.755	0.59-0.94
Yes	50	5 (10)	45 (90)	1*	-	-	-
No	7	0 (0)	7 (100)				
Maintenance OCS	57	0 [0-5]	0 [0-0]	0.229	-	-	-
Yes	5	2 (40)	3 (60)	0.056*	Si	10.89	1.12-98.75
No	52	3 (5.8)	49 (94.2)				
Baseline FEV1 (%)	57	90.6 ± 15.1	69.8 ± 22.4	0.062	-	0.96	0.90-0.99
<80	38	2 (5.3)	36 (94.7)	0.321*	-	-	-
>80	19	3 (15.8)	16 (84.2)				
Baseline ACT	43	20 [14.8-22.8]	22 [19.5-24]	1	-	-	-
Exacerbation in previous year	57	1 [0-2]	0 [0-1]	0.382	-	-	-
Yes	26	3 (11.5)	23 (88.5)	0.651*	-	-	-
No	31	2 (6.5)	29 (93.5)				
Basal blood eosinophils (cell/mcl)	57	160 [30-550]	420 [238-570]	0.172	-	-	-
Baseline IgE (IU/Ml)	45	70.2 [69-582.5]	138.4 [49.5-742]	0.852	-	-	-
Years with benralizumab	57	1 [1-2]	2 [1-3]	0.425	-	-	-
Previous BT							
Yes	19	3 (15.8)	16 (84.2)	0.321*	-	-	-
No	38	2 (5.3)	36 (94.7)				

BMI, body mass index; GERD, gastro-oesophageal reflux disease; SAHS, sleep apnoea-hypopnoea syndrome; COPD, chronic obstructive pulmonary disease; EC, eosinophilic asthma; ICS, inhaled corticosteroids; OCS, oral corticosteroids; FEV1, peak expiratory volume in the first second of forced expiration; ACT, Asthma Control Test; IgE, immunoglobulin E; BT, biological therapy. OR, Odds ratio; CI95%, 95% confidence interval.

Unsatisfactory: no more than 50% reduction in maintenance OCS dose, or complete elimination of maintenance OCS; Satisfactory: at least 50% reduction in maintenance OCS dose or elimination of maintenance OCS dose.

*Fisher's exact test
