# Supplementary material for: Impact of Anti-IL5 Therapies on Patients with Severe Uncontrolled Asthma and Possible Predictive Biomarkers of Response: A Real-Life Study
Source: Int J Mol Sci. 2023 Jan 19;24(3):2011. doi: 10.3390/ijms24032011 (PMC9917054; doi:10.3390/ijms24032011)
Supplement: Supplementary file 1 [file ijms-24-02011-s001.zip › Table S7.pdf]

Table S7: Predictors of lung function improvement at 12 months of benralizumab treatment in patients with severe uncontrolled asthma (bivariate analysis).

	**Response to improved lung function**						
Independent variable	N	Unsatisfactory	Satisfactory	p-value	Reference category	OR	$CI_{95\%}$
Age	48	56.04 ± 15.16	61.04 ± 12.87	0.223	-	-	-
Sex							
Female	30	14 (46.7)	16 (53.3)	0.823	-	-	-
Male	18	9 (50)	9 (50)				
BMI							
Underweight	0	-	-	0.300	-	-	-
Normal weight	11	7 (63.6)	4 (36.4)				
Overweight	15	5 (33.3)	10 (66.7)				
Obesity	22	11 (50)	11 (50)				
Tobacco consumption							
Non smoker	1	1 (100)	0 (0)	0.571	-	-	-
Former smoker	11	5 (45.5)	6 (54.5)				
Current smoker	36	17 (47.2)	19 (52.8)				
Previous respiratory disease							
Yes	22	11 (50)	11 (50)	0.790	-	-	-
No	26	12 (46.2)	14 (53.8)				
Polyps							
Yes	20	8 (40)	12 (60)	0.354	-	-	-
No	28	15 (53.6)	13 (46.4)				
Allergies							
Yes	29	15 (51.7)	14 (48.3)	0.514	-		
No	19	8 (42.1)	11 (57.9)				
GERD							
Yes	17	9 (52.9)	8 (47.1)	0.606	-	-	-
No	31	14 (45.2)	17 (54.8)				
SAHS							

Yes	8	5 (62.5)	3 (37.5)	0.454*	-	-	-
No	40	18 (45)	22 (55)				
COPD							
Yes	10	5 (50)	5 (50)	0.882	-	-	-
No	38	18 (47.4)	20 (52.6)				
Years with AE	48	7 [4-10.5]	6 [4-9]	0.801	-	-	-
ICS (mg/day)	48	184 [184-610]	184 [184-640]	0.712	-	-	-
Bursts of OCS per year	48	2 [1-3.5]	3 [1-4]	0.409	-	-	-
Yes	41	19 (46.3)	22 (53.7)	0.696*	-	-	-
No	7	4 (57.1)	3 (42.9)				
Maintenance OCS	48	0 [0-0]	0 [0-0]	0.489			
Yes	4	1 (25)	3 (75)	0.61*	-	-	-
No	44	22 (50)	22 (50)				
Baseline FEV1 (%)	48	81 ± 21.69	61.44 ± 20.49	0.007	-	0.96	[0.92-0.98]
<80	23	12 (36.4)	21 (63.6)	0.018	>80	4.81	[1.33-20.6]
>80	15	11 (73.3)	4 (26.7)				
Baseline ACT	27	13 [10-22]	13 [12-23]	0.771	-	-	-
Exacerbation in previous year	48	0 [1-1.5]	0 [1-1]	0.150	-	-	-
Yes	21	10 (47.6)	11 (52.4)	0.971	-	-	-
No	27	13 (48.1)	14 (51.9)				
Basal blood eosinophils (cell/mcl)	48	380 [235-505]	500 [230-650]	0.472	-	-	-
Baseline IgE (IU/Ml)	45	229 [95-988.3]	110 [45.3-522.6]	0.101	-	-	-
Years with benralizumab	48	2 [1-3]	2 [1-3]	0.466	-	-	-
Previous BT							
Yes	16	7 (13.8)	9 (56.2)	0.683	-	-	-
No	32	16 (50)	16 (50)				

BMI, body mass index; GERD, gastro-oesophageal reflux disease; SAHS, sleep apnoea-hypopnoea syndrome; COPD, chronic obstructive pulmonary disease; EC, eosinophilic asthma; ICS, inhaled corticosteroids; OCS, oral corticosteroids; FEV1, peak expiratory volume in the first second of forced expiration; ACT, Asthma Control Test; IgE, immunoglobulin E; BT, biological therapy. OR, Odds ratio; CI95%, 95% confidence interval.

Unsatisfactory: does not increase FEV1 in 10%; Satisfactory: FEV1 increase of at least 10%.

*Fisher's exact test
