# Supplementary material for: Impact of Anti-IL5 Therapies on Patients with Severe Uncontrolled Asthma and Possible Predictive Biomarkers of Response: A Real-Life Study
Source: Int J Mol Sci. 2023 Jan 19;24(3):2011. doi: 10.3390/ijms24032011 (PMC9917054; doi:10.3390/ijms24032011)
Supplement: Supplementary file 1 [file ijms-24-02011-s001.zip › Table S8.pdf]

Table S8: Predictors of exacerbation reduction at 12 months of benralizumab treatment in patients with severe uncontrolled asthma (bivariate analysis).

	Response to exacerbation reduction						
Independent variable	N	Unsatisfactory	Satisfactory	p-value	Reference category	OR	$CI_{95\%}$
Age	57	49.67 ± 5.51	58.91 ± 14.49	0.281	-	-	-
Sex							
Female	37	2 (5.4)	35 (94.6)	1*	-	-	-
Male	20	1 (5)	19 (95)				
BMI							
Underweight	-	-	-	0.586*	-	-	-
Normal weight	12	0 (0)	12 (100)				
Overweight	19	2 (10.5)	17 (89.5)				
Obesity	26	1 (3.8)	25 (96.2)				
Tobacco consumption							
Non smoker	42	0 (0)	42 (100)	0.016*	Current smoker	3.67	[1.15-16.21]
Former smoker	14	3 (21.4)	11 (78.6)				
Current smoker	1	0 (0)	1 (100)				
Previous respiratory disease							
Yes	26	3 (11.5)	23 (88.5)	0.089*	-	-	-
No	31	0 (0)	31 (100)				
Polyps							
Yes	22	0 (0)	22 (100)	0.276*	-	-	-
No	35	3 (8.6)	32 (91.4)				
Allergies							
Yes	35	2 (5.7)	33 (94.3)	1*	-	-	-
No	22	1 (4.5)	21 (95.5)				
GERD							
Yes	23	2 (8.7)	21 (91.3)	0.559*	-	-	-
No	34	1 (2.9)	33 (97.1)				
SAHS							

Yes	10	0 (0)	10 (100)	1*	-	-	-
No	47	3 (6.4)	44 (93.6)				
COPD							
Yes	11	1 (9.1)	10 (90.9)	0.481*	-	-	-
No	46	2 (4.3)	44 (95.7)				
Years with AE	57	2 [2-3.5]	7 [4-10]	0.112	-	-	-
ICS (mg/day)	57	184 [184-492]	184 [184-630]	0.836	-	-	-
Bursts of OCS per year	57	4 [2-4.5]	2 [1-4]	0.945	-	-	-
Yes	50	2 (4)	48 (96)	0.330*	-	-	-
No	7	1 (14.3)	6 (85.7)				
Maintenance OCS	57	0 [0-0]	0 [0-0]	0.997	-	-	-
Yes	5	0 (0)	5 (100)	1*	-	-	-
No	52	3 (5.8)	49 (94.2)				
Baseline FEV1 (%)	57	70 ± 25.94	71.71 ± 22.59	0.895	-	-	-
<80	38	2 (5.3)	26 (94.7)	1*	-	-	-
>80	19	1 (5.3)	18 (94.7)				
Baseline ACT	27	9 [7-11]	13 [10-16]	0.221	-	-	-
Exacerbation in previous year	57	1 [0.5-3]	0 [0-1]	0.093	-	-	-
Yes	26	2 (7.7)	24 (92.3)	0.587*	-	-	-
No	31	1 (3.2)	30 (96.8)				
Basal blood eosinophils (cell/mcl)	57	290 [190-345]	435 [230-570]	0.287	-	-	-
Baseline IgE (IU/Ml)	45	594 [592-1364.5]	112 [49.5-742.1]	0.120	-	-	-
Years with benralizumab	57	2 [1.5-2.5]	2 [1-3]	0.845		-	-
Previous BT							
Yes	19	2 (10.5)	17 (89.5)	0.255*	-	-	-
No	38	1 (2.6)	37 (97.4)				

BMI, body mass index; GERD, gastro-oesophageal reflux disease; SAHS, sleep apnoea-hypopnoea syndrome; COPD, chronic obstructive pulmonary disease; EC, eosinophilic asthma; ICS, inhaled corticosteroids; OCS, oral corticosteroids; FEV1, peak expiratory volume in the first second of forced expiration; ACT, Asthma Control Test; IgE, immunoglobulin E; BT, biological therapy. OR, Odds ratio; CI95%, 95% confidence interval.

Unsatisfactory: no reduction of 50% of exacerbations, or absence; Satisfactory: reduction of at least 50% of exacerbations or absence of exacerbations.

*Fisher's exact test
